# The Burden and Risk Factors Associated with Infectious Diseases among Refugees in a Camp for Migrants in Porto Alegre: A Cross-Sectional Survey

**DOI:** 10.5334/aogh.4242

**Published:** 2024-07-26

**Authors:** Mauricio Da Silva Roxkow Fraga, Filipe Andre Angst, James January, Agnes Madziwa, Laston Gonah, Alexandre Lazzarotto

**Affiliations:** 1 Hospital Independência de Porto Alegre; 2 Catholic University of Mozambique; 3 Department of Global Public health & Family Medicine, Faculty of Medicine and Health Sciences, University of Zimbabwe; 4 Department of Mental Health & Psychiatry Kamuzu University of Health Sciences, Malawi; 5 Department of Community Medicine, Faculty of Medicine & Health Sciences, Midlands State University, Zimbabwe

**Keywords:** infectious diseases, refugees, Brazil, risk factors

## Abstract

Refugees usually face a disproportionate burden of infectious diseases. Recently, Brazil has experienced an influx of refugees which demands the need for scaling up public health efforts to address the challenges. The research sought to study the burden and risk factors associated with infectious diseases among refugees received in the city of Porto Alegre.

This was a cross-sectional study of 261 newly arrived refugees. The study sample was predominantly composed of Venezuelans (50.6%) and Haitians (44%), male (146: 56.7%), single (30.7%), with an average age of 33.38 (± 7.30) years. The average schooling was 10.42 (± 2.09) years. Diseases with the highest prevalence were influenza, whooping cough, diphtheria, and tuberculosis. There was significant association between the country of origin and presence of symptoms for infectious and contagious diseases, which warrants targeted interventions for reducing the incidence of these diseases among refugees in Brazil.

## Introduction

The act of migrating has been present since the beginning of mankind, when humans were still nomadic, constantly moving from place to place in search of better living conditions and survival. Such movements may have inevitably contributed to the spread of infectious diseases across the globe [1]. According to the World Migration Report of 2020, it is estimated that there were approximately 281 million international migrants worldwide in 2020, which constituted 3.6% of the global population [2]. The same report also stated that in 2018, the global refugee population stood at 25.9 million. The data released by the United Nations High Commissioner for Refugees (UNHCR) revealed a sharp increase in number of immigrants, globally: in 2018, 70.8 million people were forced to leave their homes, with more than half of this population under 18 years of age. This figure is about 22 million more than the previous decade, with more than half of this population under 18 years of age [3].

In most cases, these refugees are displaced due to hunger; unfavorable political conditions; existence of armed conflicts; and racial, social, religious, and even environmental discrimination. In this context, Brazil became the destination for people fleeing the danger caused by wars and other world conflicts [4]. In 2018, Brazil received 41,915 asylum requests, according to the National Committee for Refugees [5], representing 10,000 more than in 2017. Considering nationality, the largest proportion of refugees were made up of Venezuelans (52.75%), Cubans (7.01%), Haitians (6.97%), Angolans (6.01%), and Chinese (4.32%) [5].

The growing demand for foreigners to enter Brazil has led to concerns about the fragility of the government structures responsible for receiving and welcoming these individuals. There are important public health measures that must be instituted, including screening for infectious diseases and offering the necessary health interventions. Previous studies have reported poor health outcomes among refugees and immigrants in Brazil [6]. The additional strain on the healthcare system as a result of these mass movements adds to the already existing concerns over re-emerging diseases caused by environmental changes. These diseases include Chikungunya, yellow fever, Zika virus, dengue fever, and leishmaniasis [7]. There is also evidence to show that migrants in Brazil are disproportionately affected by diseases such as latent tuberculosis [8], HIV/AIDS, leishmaniasis, and malaria [9]. Low childhood immunization coverage was also found to increase the risk of infectious diseases such as tuberculosis, HIV, measles, COVID-19, and hepatitis C [10, 11]. Stephenson [12] highlighted measles as a serious concern due to the usually low vaccination coverage among refugees. To overcome these problems, some non-governmental institutions carry out voluntary actions with the objective of engaging newcomers regarding accessibility to essential services.

Despite these efforts, important gaps are observed regarding the health conditions of refugees received in Brazil. Most of these health challenges center on access to adequate sanitation and health care in the country of origin. Additionally, there is lack of a holistic view of the newly arrived individuals, who are usually composed of diverse cultures regarding the understanding of health and disease. However, public health policies, which are covered by the policies that govern the Unified Health System – SUS, do not address the gaps and realities that are related to migrants’ health [13].

In Porto Alegre, there is the Italo-Brazilian Centre for Assistance and Instruction to Migration (CIBAI), founded on April 16, 1958, which proposes to elaborate and develop humanitarian actions of the Pompeia Mission that focus its work on migrations, in addition to being a member of the Forum Permanent Human Mobility and COMIRAT-RS. CIBAI works with refugees, helping with the regularization of documents, insertion into the labor market, and the teaching of Portuguese. It is made up of its own team and volunteers from areas such as law, psychology, nursing, letters, and social assistance, which in 2017 alone served more than 7,000 immigrants. Even if the refugees are welcomed through specific regional health policies linked to the Unified Health System, the health statuses of newly arrived foreigners is usually unknown.

Given the above highlighted needs on the health of refugees, this study aimed to identify the burden and risk factors associated with infectious diseases among the refugees received at a reference centre for migration in Alegre Port (Rio Grande do Sul-Brazil). The aim is to contribute to the elaboration and application of a protocol to assist refugees that identifies the socio-demographic aspects, illness-related reports, pain intensity, and risk factors for infectious diseases, thus enabling the referral for assistance (treatment and immunization) in the healthcare system.

## Methods

A cross-sectional study, approved by the Research Ethics Committee (CAAE: 04717718.8.0000.5307) was conducted. The study participants were immigrants in refugee situations who have recently arrived in Brazil, aged 18 years or over, of both sexes. They were invited with of aid of interpreters in English, French, Creole, and Spanish (according to their native languages). An Informed Consent Form (TCLE) (translated into each language) was signed by every participant. The study location was a reference centre for migrations located in the city of Porto Alegre (Rio Grande do Sul-Brazil). Data collection took place from January to July 2019.

### Sampling

The study participants were selected from consultation registers using systematic random sampling. Yamane’s (1973) formula was used to calculate the sample size. Considering total population of 7,075 (total number of consultations done at the centre of refugees in 2018) and establishing a confidence level of 90% with a margin of error of 5%, a sample size of 261 participants was obtained.


\[
n = \frac{N}{{1 + N{{\left( e \right)}^2}}}
\]

*n* = Sample size

*N* = Population size

*e* = margin of error

\(n = \frac{{7075}}{{1 + 7075{{\left( {0.05} \right)}^2}}} = 261,02702702{\mkern 1mu}  \simeq 261\) participants

## Data Collection

For data collection, the Health Admission Protocol (PAS) was developed and applied, which was tested through blind inter-judge evaluation and qualified through a pilot study. The PAS consists of a script of questions about the socio-demographic profile, presence and severity of pain, perception of quality of life, body mass index (BMI), and risk factors for developing infectious/contagious diseases.

For infectious/contagious diseases, reports of the presence of up to four symptoms present in the retroactive period of 90 days for pertussis, influenza, diphtheria, mumps, tetanus, herpes zoster, yellow fever, hepatitis, syphilis, and tuberculosis were used. It should be noted that the greater the number of symptoms, the greater the probability that the participant is infected.

The infectious/contagious diseases mentioned in the PAS were selected from the public health notebooks developed by the Ministry of Health of Brazil on the main diseases present in the country in 2017, and the four main defining characteristics for each of them were identified.

Data collection was performed according to the following steps: (a) explanation of the research with the aid of an interpreter, (b) invitation and signing of the Informed Consent Form translated into three different languages and delivered in the participant’s native language, and (c) explanation and application of the protocol with the assistance of an interpreter. Participants who reported latent symptoms or the presence of at least three risk factors for any of the diseases present in the PAS were instructed to seek care in the primary healthcare network of reference and, consequently, the employees of the migration centre were informed about these participants’ health conditions.

Quantitative variables were described by mean, standard deviation, and categorical variables, described by absolute and relative frequencies. To verify the differences between the study variables, the Student’s *t-*test for independent samples was used. To verify the associations, Fisher’s exact test was performed (*p* < 0.05). The statistical treatment was performed using the SPSS package, version 24.0 for Windows.

## Results

Table 1 shows the frequency and percentage distribution of participants’ socio-demographic characteristics such as sex, age, marital status and country of origin. The study sample of 261 participants was predominantly composed of Venezuelans (50.6%), Haitians (44%), and males (146:56.7%). The mean age was 33.38 (± 7.30) and the education level was 10.42 (± 2.09) years. The predominant marital status was married (46.7%) and the prevalent religion was Catholic (59.8%).

**Table 1:** Socio-demographic profile of participants.

VARIABLES	*N* = 261
**Age (a)**	33.38 ± 7.30
**Sex**	
Male	148 (56.7%)
Female	113 (43.3%)
**Marital Status**	
Married	122 (46.7%)
Single	80 (30.7%)
Widow	52 (19.9%)
Other	7 (2.7%)
**Country of Origin**	
Venezuela	132 (50.6%)
Haiti	115 (44%)
Others	14 (5.4%)
**Years of Study**	10.42 ± 2.09
**Church denomination**	
Catholic	156 (59.8%)
Other	65 (24.9%)
Evangelic	40 (15.3%)
**Language**	
Spanish	143 (54.8%)
French	109 (41.8%)
Creole	89 (64.1%)
English	8 (3.1%)

** Multiple choice response because, in the language variable, participants reported fluency in more than one option**.**

(a) Results expressed as mean ± standard deviation. Other results through frequency analysis.

On the family structure, 79.7% of the participants reported having no relatives in Brazil. Regarding the number of children, 42.2% of the participants had two or more, and 61.3% had left their children with relatives in their country of origin. Most participants (87%) had no formal occupation or income-generating activity at the time of the study. Participants reported that lack of formal occupation or income-generating projects was distressing, and they cited it as the main reason for choosing Brazil as their destination. In their country of origin, some of the participants reported having a paid occupation, mainly in the commercial sector (31.8%), industrial sector (12.6%), and rural work (11.9%).

**Health assessment**

Health assessment revealed that 51.1% of the participants considered they were in good health and 22.6% in excellent health. Results on quality of life were favorable: 51.8% reported having a good quality of life and 17.6% reported having a very good quality of life. The BMI identified that 56% of participants were eutrophic and 41.4% overweight. Regarding the presence of pain (0–10 in intensity) there was a predominance of zero value in 67.8% of the participants, indicating a favorable physical condition for the performance of physical and work activities.

**Risk factors for infectious/contagious diseases**

**Figure 1:**
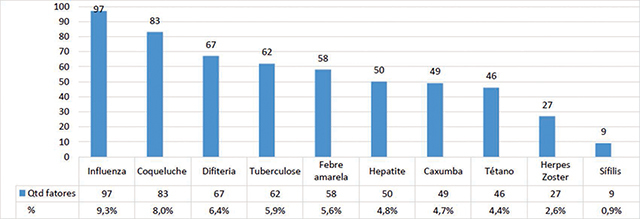
Prevalence of infectious diseases among refugees at Porto Alegre.

Regarding the risk factors for infectious/contagious diseases, Figure 1 summarizes the prevalence of factors associated with the pathology present in the study sample within 90 days prior to the date of data collection, regardless of the individual’s arrival in Brazil.

The diseases with the highest prevalence were influenza (9.3%), followed by pertussis (8%), diphtheria (6.4%), and tuberculosis (5.9%). Regarding the frequency of symptoms for infectious/contagious diseases, influenza had 39 reports of muscle pain (14.9%), followed by 24 reports of nasal discharge (9.2%), 17 reports of fever (6.5%), and 17 reports of weakness (6.5%). For whooping cough, there was a predominance in reporting of frequent sneezing, with 38 participants (14.6%), followed by nasal discharge for 22 (8.4%), fever (6.5%), and watery eyes (2.3%). For diphtheria, 28 participants (5.4%) reported throat pain and hoarseness, 17 participants (6.6%) reported fever, followed by 5.4% nasal discharge and 1.1% difficulty in speaking and swallowing. For tuberculosis symptoms, there was a predominance of cachexia with 20 (7.7%) reports, followed by weakness with 16 (6.9%), night sweating with 5.7%, and coughing with the presence of blood with 3.4% of the reports.

The results showed a significant association between the country of origin and the presence of symptoms for infectious and contagious diseases. Table 2 shows significant correlations of risk factors for Haitians, even with the predominance of Venezuelans in the study sample.

**Table 2:** Association of risk factors for influenza, pertussis, diphtheria and tuberculosis with the other study variables.

**Influenza**	Muscle Pain			Fever			Nasal Discharge			Weakness		
	No	Yes	*p*	No	Yes	*P*	No	Yes	*p*	No	Yes	*p*
**Age (a)**	33.51 ± 7.43	33.59 ± 6.56	00.85	33.42 ± 7.41	33.88 ± 5.6	00.71	33.07 ± 6.62	36.45 ± 11.90	00.18	36.45 ± 7.24	32.52 ± 8.6	00.66
**Sex (b)**			00.2			00.46			00.18			00.17
Female	99 (44.6%)	14 (35.9%)		105 (43%)	8 (47.1%)		100 (42.2)	13 (54.2%)		108 (44.3%)	5 (29.4%)	
Male	123 (55.4%)	25 (64.1%)		139 (57%)	9 (52.9%)		137 (57.8)	11 (45.8%)		136 (55.7%)	12 (70.6%)	
**Country of origin**			00.04**			00.03**			00.23			00.04**
Haiti	93 (41.9%)	22 (56.4%)		101 (41.4)	14 (82.4)		104 (43.9)	11 (45.8%)		102 (41.8%)	13 (76.5%)	
Venezuela	93 (41.9%)	13 (33.3%)		130 (53.3)	2 (11.8%)		122 (51.5)	10 (41.7%)		130 (5.3%)	2 (11.8%)	
Others	10 (4.5%)	4 (10.3%)		13 (5.3%)	1 (5.9%)		11 (4.6%)	3 (12.5%)		12 (4.9%)	2 (11.8%)	
**Pertussis**	Frequent Sneezing			Fever			Nasal Discharge			Watery Eyes		
	No	Yes	*p*	No	Yes	*P*	No	Yes	*p*	No	Yes	*p*
**Age (a)**	33.12 ± 6.81	34.92 ± 9.65	00.27	33.42 ± 7.41	32.88 ± 5.6	00.71	33.10 ± 6.61	36.41 ± 12.39	00.23	33.36 ± 7.34	4.33 ± 5.27	00.67
**Sex (b)**			00.14			00.46			00.18			00.47
Female	100 (44.8%)	13 (34.2%)		105 (43%)	8 (47.1%)		101 (42.3)	12 (54.5%)		111 (43.5%)	2 (33.3%)	
Male	123 (55.2%)	25 (65.8%)		139 (57%)	9 (52.9%)		138 (57.7)	10 (45.5%)		144 (56.5%)	4 (66.7%)	
**Country of origin**			00.1			00.03**			00.12			00.06
Haiti	95 (42.6%)	20 (52.6%)		101 (41.54)	14 (82.4)		104 (43.5)	11 (50%)		112 (43.9%)	3 (50%)	
Venezuela	118 (52.9%)	14 (36.8%)		130 (53.3)	2 (11.8%)		124 (51.9)	8 (36.4%)		131 (51.4%)	1 (16.7%)	
Others	10 (4.5%)	4 (10.5%)		13 (5.3%)	1 (5.9%)		11 (4.6%)	3 (13.6%)		12 (4.7%)	2 (33.3%)	
**Diphtheria**	Frequent Sneezing			Fever			Nasal Discharge			Watery Eyes		
	No	Yes	*p*	No	Yes	*p*	No	Yes	*p*	No	Yes	*p*
**Age (a)**	33.12 ± 6.81	34.92 ± 9.65	00.27	33.42 ± 7.41	32.88 ± 5.56	0.71	33.10 ± 6.61	36.41 ± 12.39	00.23	33.36 ± 7.34	4.33 ± 5.27	00.67
**Sex (b)**			00.14			00.46			00.18			00.47
Female	100 (44.8%)	13 (34.2%)		105 (43%)	8 (47.1%)		101 (42.3)	12 (54.5%)		111 (43.5%)	2 (33.3%)	
Male	123 (55.2%)	25 (65.8%)		139 (57%)	9 (52.9%)		138 (57.7)	10 (45.5%)		144 (56.5%)	4 (66.7%)	
**Country of origin**			00.1			00.03**			00.12			00.06
Haiti	95 (42.6%)	20 (52.6%)		101 (41.54)	14 (82.4%)		104 (43.5)	11 (50%)		112 (43.9%)	3 (50%)	
Venezuela	118 (52.9%)	14 (36.8%)		130 (53.3)	2 (11.8%)		124 (51.9)	8 (36.4%)		131 (51.4%)	1 (16.7%)	
Others	10 (4.5%)	4 (10.5%)		13 (5.3%)	1 (5.9%)		11 (4.6%)	3 (13.6%)		12 (4.7%)	2 (33.3%)	
**Tuberculosis**	Muscle Pain			Fever			Nasal Discharge			Weakness		
	No	Yes	*p*	No	Yes	*p*	No	Yes	*p*	No	Yes	*p*
**Age (a)**	33.39 ± 7.39	33.22 ± 4.29		33.61 ± 7.42	30.60 ± 4.1		33.42 ± 7.40	32.80 ± 5.3		33.44 ± 7.38	32.61 ± 6.4	
**Sex (b)**			00.17			00.52			00.14			00.26
Female	111 (44%)	2 (22.2%)		104 (43.2)	9 (45%)		109 (44.3%)	4 (26.7%)		107 (44%)	6 (33.3%)	
Male	141 (56%)	7 (77.8%)		137 (58.8)	11 (55%)		137 (55.7%)	11 (73%)		136 (56%)	12 (66.7)	
**Country of origin**			00.11			00.04**			00.01**			00.01**
Haiti	108 (42.9%)	7 (77.8%)		101 (41.9)	14 (70%)		108 (43.9%)	7 (46.7%)		102 (42%)	13 (72.2)	
Venezuela	130 (5.6%)	2 (22.2%)		127 (52.7)	5 (25%)		129 (52.4%)	3 (20%)		129 (53.1%)	3 (16.7%)	
Others	14 (5.6%)	0 (0%)		13 (5.4%)	1 (5%)		9 (3.7%)	5 (33%)		12 (4.9%)	2 (11.1%)	

(a) *t-*test for independent samples.

(b) Fischer’s exact test.

** Significance level 0.05.

## Discussion

This study aimed to contribute to literature on the burden and risk factors associated with of infectious/contagious diseases prevalent among refugee and migrant populations. Specifically, factors associated with infectious diseases among refugees received in the city of Porto Alegre were explored.

The study sample comprised mostly of Venezuelans, which may be explained in part by the current instability in the country, which is forcing individuals to migrate to safer countries. Previous studies have also revealed that this crisis has led to increases in infectious diseases in neighboring countries such as Brazil and Colombia [14]. Such findings concur with the results of this paper and raise the urgent need for improved border health services. Currently, Brazil is the country with the largest migratory flow of the Venezuelan population, and the arrival of migrants in 2018 formed the largest South American humanitarian corridor [15]. The result of this increase in the entry of migrants was the overload of public services in the regions, causing delays in the processes of insertion of the population into the country, which had as a characteristic poor health rates such as malnutrition in children in the range of 17%, one of the worst in the Americas [5].

Interestingly, half of our respondents reported a favorable quality of life. The World Health Organization (WHO) conceptualizes quality of life as the perception that an individual has about their position in life, in contexts such as culture, values, goals, expectations, standards, and concerns. Thus, quality of life indicates the level of basic and supplementary conditions of the human being, which involve individual aspects (physical, mental, psychological, and emotional well-being) and collective (relationships, family, health, and work) that directly affect the human being’s life [16, 17]. Such findings are encouraging, as individuals who perceive themselves in a positive light are more likely to seek health services and are less likely to suffer from other conditions such as depression which may weaken their immune systems.

Our results also showed that almost half of the respondents were classified as overweight. The control and reduction of obesity is one of the greatest challenges worldwide, despite campaigns to raise awareness about overweight (BMI > 25) and obesity (BMI > 30) that increase significantly every year. These factors are directly linked to arterial hypertension, frequently detected in Brazilian studies [18]. Thus, in addition to screening for infectious diseases among these vulnerable populations, health promotion interventions aimed at reducing non-communicable diseases such as hypertension are needed.

From the study sample, the diseases with the highest prevalence were influenza, pertussis, diphtheria, and tuberculosis. It should be noted that infectious and respiratory diseases are the main causes of hospitalization in Brazil [19]. These results, therefore, are relevant insofar as they support the need for measures aimed at reducing hospitalizations for specific preventable pathologies, especially in the north and northeast regions, regions with the highest volume of foreign population inflows [20, 21]. The study presented the participants’ reports on the presence of symptoms in the period of 90 days prior to the date of data collection. For the diseases studied, this period represents a high risk for transmission. Infectious/contagious diseases such as influenza and tuberculosis present a risk of multi-resistance lasting longer in the latency period in the transmitter’s body, thus increasing the incidence of new cases [22].

Based on the results of the study, the fragility of the health of the Haitians received in Porto Alegre became evident. Haiti is located on the second-largest island in the Caribbean. In January 2010, an earthquake of catastrophic proportions caused the destruction of 80% of the buildings in the country’s capital, the most populous city [23]. Even after several years, the health situation in Haiti has the lowest health indicators in America, as result of political and economic degradation contributing to the lack of basic sanitation and the fragility of its health system, resulting in high mortality and morbidity rates of diseases already eradicated or controlled in other countries [24]. In this context, our study showed strong correlation between the most reported common symptom, fever, in the population of Haitians, and the four prevalent pathologies. In addition, the weakness symptom was the most present for influenza. However, tuberculosis was not prevalent in this population.

As a result of this study, important gaps were observed in the inclusion and integration of migrants into the Brazilian health system, where measures of active searches for newcomers by the state are not identified; there is only the availability of an SUS not yet presented to the migrant.

The adaptation of an individual or group from another ethnic group in Brazilian territory requires specific policies with more effective action planning, consisting of inclusion programs focused on the search and an even greater effort on the part of agents working in refugee reception. This fact becomes a challenge with important consequences, such as the marginalization of access to primary healthcare services and the spread of infectious/contagious diseases among the general population.

The lack of healthy conditions for refugees and the weaknesses of the entities that receive this population are problems of great concern, as it is considered a fragile and precarious reality. In refugees settlements usually there are poor water and sanitation conditions, overcrowding, and poor housing that increases their risks of contracting infectious diseases. One of the relevant aspects is the age group, predominantly composed of young adults who seek immediate inclusion into Brazilian society through an opportunity in the formal or informal labor market, a factor that further aggravates the monitoring of the health of this population. International migrations bring about changes and new dynamics in the societies of origin and destination. It is a complex phenomenon that places demands on different areas of life and knowledge, including health.

In addition, risk factors associated with infectious diseases identified include educational level and country of origin. This is in consistent with a study by Ayele et al. [25] that highlighted risk factors for hepatitis B and C viruses among refugees in Gambella, Ethiopia. The results of this study compliments the finding of another study by Kamali et al. [26] which highlighted that age and sex are associated with risk of infection. This study also added other factors like comorbidity, family history, type of shelter, and unhygienic practices; participants who visited traditional healers and history of surgical operation are significantly associated with having an infectious disease.

Ergönül et al. [27] highlighted how temporary shelter increases the risk of respiratory tract infections. In addition, women were found at higher risk of contracting sexually transmitted infections. Also, hospital-acquired infections were reported among the refugees.

## Study Limitations

Our study had several limitations. First, the sample size used was small, which makes it difficult to generalize our findings on a large scale. Second, the cross-sectional nature of the study makes it difficult to infer causality. Third, there was a possibility of recall bias among our respondents, as they were asked to remember how they felt in the past 90 days. Considering these limitations, further studies could utilize large sample sizes and possibly include a longitudinal design so as to ascertain whether these vulnerable populations continue to be at increased risk for poor health outcomes.

## Conclusion

This study showed that there is increased risk for infectious diseases among sub-populations of refugees in Brazil. It is therefore imperative to design and implement targeted public-health interventions aimed at reducing the incidence of infectious diseases. In addition to improved border health services, there is need the need for educational campaigns to increase access to health information among refugees and ensure they are vaccinated. The Health Admission Protocol (PAS) is sought to assess the health of the newly arrived refugee population through an easy-to-apply instrument with the proposal to provide, through its results, an overview of the health condition and the presence of important symptoms for the elaboration of a primary diagnosis of diseases with a high rate of contagion and transmission, becoming an important tool for primary, secondary, and tertiary health care.

Noori et al. [28] emphasized the need to strengthen the evidence base and to advocate for universal access to health care for all refugees and asylum seekers. The health services must be voluntary, confidential, and non-stigmatizing; screening must be affordable; and refugees and asylum seekers must be on equal footing with nationals.
